# A Comprehensive Motion Estimation Technique for the Improvement of EIS Methods Based on the SURF Algorithm and Kalman Filter

**DOI:** 10.3390/s16040486

**Published:** 2016-04-07

**Authors:** Xuemin Cheng, Qun Hao, Mengdi Xie

**Affiliations:** 1 Graduate School at Shenzhen, Tsinghua University, Shenzhen 518055, China; cheng-xm@mail.tsinghua.edu.cn (X.C.); xie.mengdi12@alum.sz.tsinghua.edu.cn (M.X.); 2 Beijing Key Lab. for Precision Optoelectronic Measurement Instrument and Technology, School of Optoelectronics, Beijing Institute of Technology, Beijing 100081, China

**Keywords:** vehicle platform, digital stabilization, SURF, RANSAC, cascade parameters, Kalman filter

## Abstract

Video stabilization is an important technology for removing undesired motion in videos. This paper presents a comprehensive motion estimation method for electronic image stabilization techniques, integrating the speeded up robust features (SURF) algorithm, modified random sample consensus (RANSAC), and the Kalman filter, and also taking camera scaling and conventional camera translation and rotation into full consideration. Using SURF in sub-pixel space, feature points were located and then matched. The false matched points were removed by modified RANSAC. Global motion was estimated by using the feature points and modified cascading parameters, which reduced the accumulated errors in a series of frames and improved the peak signal to noise ratio (PSNR) by 8.2 dB. A specific Kalman filter model was established by considering the movement and scaling of scenes. Finally, video stabilization was achieved with filtered motion parameters using the modified adjacent frame compensation. The experimental results proved that the target images were stabilized even when the vibrating amplitudes of the video become increasingly large.

## 1. Introduction

Photographic jitter, caused by the vibration of a moving camera, often produces undesirable effects, which video stabilization methods are designed to mitigate or eliminate. Image stabilization technologies such as mechanical image stabilization (MIS), optical image stabilization (OIS) [1], and most recently electronic image stabilization (EIS) [2] are widely applied in areas such as camera capture, vehicle monitoring, and airborne and shipboard observations. Mechanical and optical image stabilization methods usually involve adjusting the spatial positions of an element or group of elements. Compared with MIS and OIS, EIS as a software-based approach has the advantage of lower cost and easier integration, though there are limitations to the software’s accuracy and speed and the hardware’s image performance. EIS may also be used as the fine stabilization frame in a coarse/fine combination two-level stabilization approach [3,4,5]. For an in-vehicle camera, EIS is relatively cost-efficient and may be the best option.

Research into this approach has focused on developing accurate, high-speed EIS with image blur alleviation. EIS systems generally have three main components: Global motion estimation, motion smoothing, and motion compensation [6,7,8,9,10,11,12,13,14,15,16,17,18,19]. These involve: (1) extracting high-precision features and obtaining precise positioning points; (2) separating the camera’s intentional scanning movement and random noise vibration using discrete filters based on motion estimation results; (3) recovering the original frames using the smoothing parameters in the process of motion compensation. Block matching is primarily used for feature extraction and detecting the motion vector of a moving frame [6,7]. However, block matching cannot deal with scalar and rotational movement without excess computation time; in these cases, local gradient operators such as Harris and bit-plane operators are preferred [8,9]. Moreover, a moving frame has the characteristics of a dynamic scene, which may dictate a multi-resolution design. To address this issue, adaptive pyramid algorithms with a probabilistic direction have been proposed to respond to disturbances [10,11,12,13,14]. Another issue in EIS design is position-varying vibration caused by the shaking of in-vehicle cameras. Numerous suppression techniques [15,16,17,18,19,20,21,22] such as Kalman filtering, tracking differential filtering, Wiener filtering, cascaded calculation and oriented descriptors have been employed to increase the robustness of the motion estimation system against vibrations. Despite the aforementioned research, two aspects of this approach have not been comprehensively addressed: (1) the extraction and suppression techniques were designed for a balance between speed and accuracy rather than with attention to conceptual design; (2) the techniques were tested mostly for improvement of image quality. The techniques used in EIS have been developed to handle features in some different pixel spaces, but cannot capture the full complexity of features in all different pixel spaces. In addition, previous studies evaluated the effect of video stabilization by the signal-to-noise ratio rather than suppression variabilities. Moreover, the signal-to-noise ratio is affected by hardware limitations when discussing object motion. The main purpose of this paper is to address these two deficiencies in the research.

In this paper, a comprehensive motion estimation method for electronic image stabilization is realized that integrates the SURF algorithm, modified RANSAC, and the Kalman filter, taking camera scaling and conventional camera translation and rotation into full consideration. The SURF algorithm is selected for feature point extraction and description, as it is scale- and rotation-invariant offering more robustness for scaling, rotation, and translation parameters. Previous discussions [23,24,25] have shown that in the pixel space of one pixel, the Oriented BRIEF (Orb) method performs better than SURF in terms of speed and accuracy; with a smaller pixel space, e.g., 0.1 pixels, different result variabilities are observed and considered in the design. In this paper, we consider control of accuracy as well as suppression variabilities. To achieve this goal, the SURF algorithm is selected for first consideration for accuracy improvement in a comparative discussion concerning sub-pixel space, as the speed of feature point extraction will rely on the embedded system structure in future work. The exclusion of false matched points is realized with modified RANSAC. In addition, linear correlation coefficients for trajectory curves are calculated to assess the efficiency of vibration suppression in an experiment using mobile in-vehicle videos. They also are used for the selection of reference frames in image sequences, when considering the effect of cascaded parameters in the Kalman filtering process.

In the application of in-vehicle EIS methods, these techniques can be integrated in the static or mobile system, depending on the motion state of the vehicle. In this paper, the scheme proposed is applied to mobile in-vehicle videos. The comprehensive technique for video stabilization is a cascade-correlation algorithm applied to two-dimensional digital signal processing. It possesses the inherent characteristics of structural pipeline models, which make it suitable for mapping onto FPGA substrates and available for a miniaturized embedded system. Here, we focus only on the cascade-correlation algorithm and its effect. The architecture design of the embedded system is not discussed.

## 2. Materials and Methods

### 2.1. The Scheme of the EIS Method

**Figure 1:**
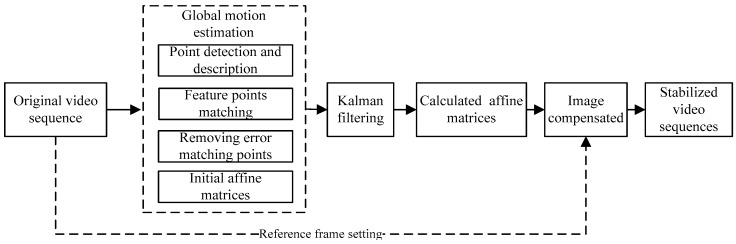
Block graph of the stabilization process.

The scheme in this paper is designed as shown in Figure 1. In the process of global motion estimation, the feature points in the original videos are distinguished and the affine matrices are determined by using the values of matched feature points in the previous and current frames. The image motion is then compensated with the affine matrices.

**Figure 2:**
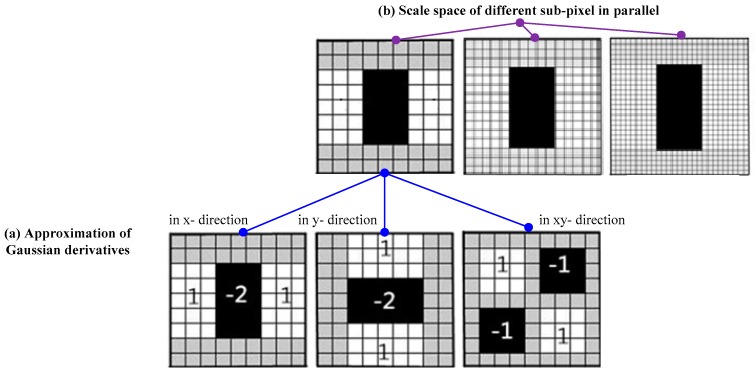
(**a**) Approximation of Gaussian derivatives using box filters; (**b**) scale space of different sub-pixels in parallel.

First, in the SURF algorithm, the potential feature points are distinguished with a Hessian matrix and non-maximum suppression, in which box filters are used to approximate Gaussian derivatives to simplify the Hessian matrix calculation, as shown in Figure 2a. At the same time, the original image is transformed into coordinates using the multi-resolution pyramid technique. Thus a copy of the image is obtained with the same size but with reduced pixel bandwidth, achieving the space of different sub-pixel scale in parallel, as shown in Figure 2b. The feature points are selected by locating extreme points by means of the gradient value around the points. The Haar wavelet responses in both x- and y-directions around the point of interest are then computed to set a multidimensional vector as the SURF feature descriptor.

Second, the affine model between two adjacent *n*th and (n−1)th frames is described for the process of global motion estimation. $I_{n}(p)=\left(x_{n},y_{n}\right)$ and $I_{n-1}(p)=\left(x_{n-1},y_{n-1}\right)$ refer to the corresponding points, as shown in Formula (1):

(1) $$I_{n}\left(p\right)=A_{n-1}I_{n-1}\left(p\right)+B_{n-1}$$

**Figure 3:**
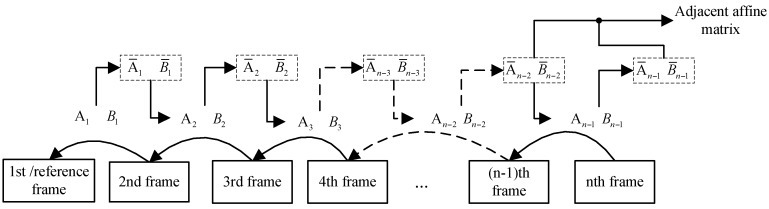
Adjacent affine model defined by using cascading parameters in the video sequences.

The cascading parameters are defined in Formula (2), in which $A_{n-1}=\left[\begin{array}{cc} a_{n-1} & b_{n-1}\\ c_{n-1} & d_{n-1} \end{array}\right]$ refers to rotation and scaling, and $B_{n-1}=\left[\begin{array}{c} e_{n-1}\\ f_{n-1} \end{array}\right]$ to translation. Figure 3 shows how the adjacent affine matrix is achieved subsequent to the previous frame through cascading parameters, which describe the current frame’s motion relative to the reference frame. To reduce accumulated errors in a series of frames, the modified cascading parameters ${\bar{A}}_{n-1}$ and ${\bar{B}}_{n-1}$ are deduced from the adjacent cascading parameters, as defined in Equation (2):

(2) $$\begin{array}{ll} I_{n}\left(p\right)=A_{n-1}I_{n-1}\left(p\right)+ & B_{n-1}=\cdots ={\bar{A}}_{n-1}I_{1}\left(p\right)+{\bar{B}}_{n-1}\\ & {\bar{A}}_{n-1}=\prod_{i=1}^{n-1}A_{i}\left(n\geq 2\right)\\ & {\bar{B}}_{n-1}=A_{n-1}{\bar{B}}_{n-2}+B_{n-1}\left(n\geq 0,{\bar{B}}_{0}=0\right) \end{array}$$

Third, the removal of false matching points with the modified RANSAC method is realized. Iteratively, the foreground and background scenes are distinguished and the false matching points are removed by using a local optimal motion estimation model, along with matched points in the foreground. The data are thus preprocessed to remove noise, or false matching points.

**Figure 4:**
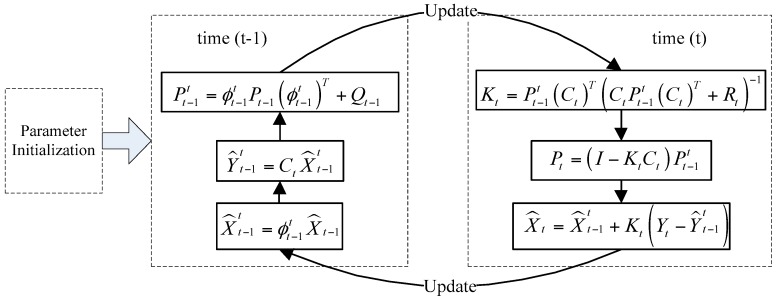
Kalman filtering scheme.

The Kalman filter is then applied for removal of high-frequency vibration from the trajectory curves by distinguishing the camera’s intentional motion from vibrations or jitters. The tracking of the estimated state and the variance or uncertainty is applied by the Kalman filter model [20], as shown in Figure 4*.* State variable ${\hat{X}}_{t-1}^{t}$ at time (t−1) is estimated by using the Kalman state transition model $\phi_{t-1}^{t}$ and the filtered result ${\hat{X}}_{t-1}$ at time (t−1). The observation data ${\hat{Y}}_{t-1}^{t}$ are estimated by using ${\hat{X}}_{t-1}^{t}$ and the observation model $C_{t}$. The error variance forecast $P_{t-1}^{t}$ at time *t* is defined by using $\phi_{t-1}^{t}$, the error variance $P_{t-1}$ at time (t−1) and the process noise covariance matrix $Q_{t-1}$. At time *t*, the Kalman gain $K_{t}$ is then calculated by $P_{t-1}^{t}$, $C_{t}$, and the observation noise covariance matrix $R_{t}$. The error variance $P_{t}$ at time *t* is defined by using the unit matrix I, $K_{t}$, $C_{t}$ and $P_{t-1}^{t}$. At time *t*, the filtered or expected state ${\hat{X}}_{t}$ is then updated using ${\hat{X}}_{t-1}^{t}$, $K_{t}$, ${\hat{Y}}_{t-1}^{t}$, and the actual observation $Y_{t}$ at time *t*. The parameters of each frame in the videos are then recursively calculated.

Finally, the motion is compensated by using the filtered affine matrices ${\hat{A}}_{n}$ and ${\hat{B}}_{n}$ at frame *n*. The position compensation parameters $A_{n-1}^{c}={\hat{A}}_{n-1}{\left({\bar{A}}_{n-1}\right)}^{-1}$ and $B_{n-1}^{c}=-{\hat{A}}_{n-1}{\left({\bar{A}}_{n-1}\right)}^{-1}{\bar{B}}_{n-1}+{\hat{B}}_{n-1}$ are applied according to Equation (3):

(3) $${\hat{I}}_{n}\left(p\right)=A_{n-1}^{c}I_{n}\left(p\right)+B_{n-1}^{c}$$

where $I_{n}\left(p\right)$ and ${\hat{I}}_{n}\left(p\right)$ refer to the initial and stabilized *n*th frames, respectively, and the modified cascading parameters ${\bar{A}}_{n-1}$ and ${\bar{B}}_{n-1}$ are deduced for smaller accumulated errors by using the selected reference frame (the first frame in a continuous sequence) instead of the adjacent frame. It is then refreshed periodically. The current frame is set as the reference frame when the suppression variabilities in the scenes, e.g., linear correlation coefficients for the trajectory curves (stated in Section 2.3), are smaller than certain values, *i.e.*, 0.9, for the consideration of the efficiency assessment of vibration suppression.

### 2.2. Selection of Feature Point Detection Algorithms

In this section, the SURF algorithm is investigated in sub-pixel space and compared with two widely used methods, the Scale Invariant Feature Transform (SIFT) [23,24] and Orb [25] algorithms, to find the best algorithm for fast and accurate feature point detection.

**Figure 5:**
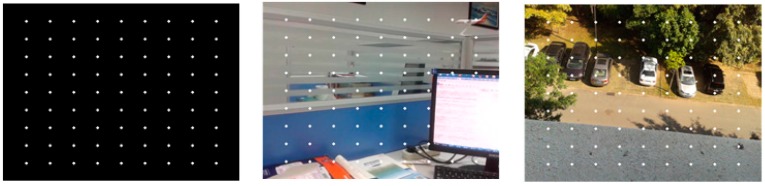
Three images with feature points placed on them.

**Figure 6:**
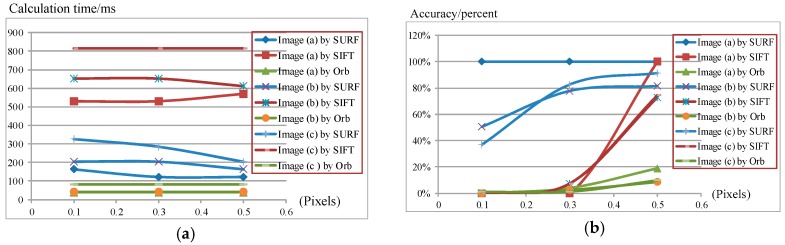
Testing results for (**a**) speed and (**b**) accuracy in feature point detection.

**Figure 7:**
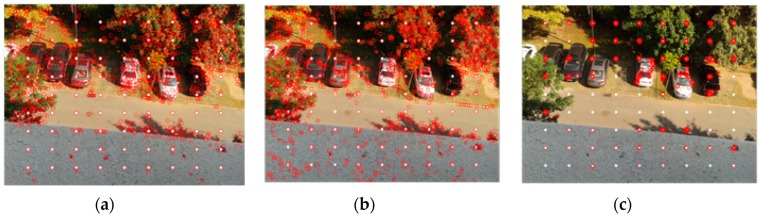
Outdoor scene results at 0.1 pixels, using the (**a**) SURF; (**b**) SIFT; and (**c**) Orb algorithms.

**Figure 8:**
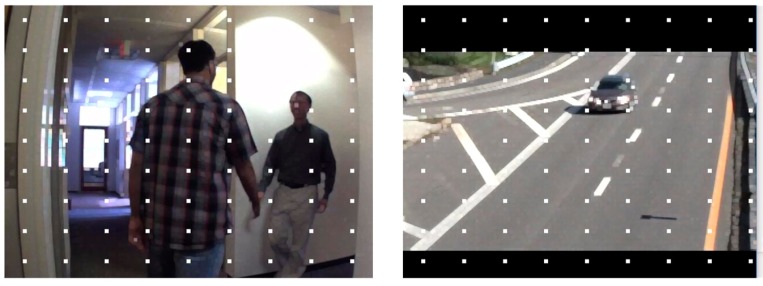
Images in two sample sequences with feature points placed on them.

**Figure 9:**
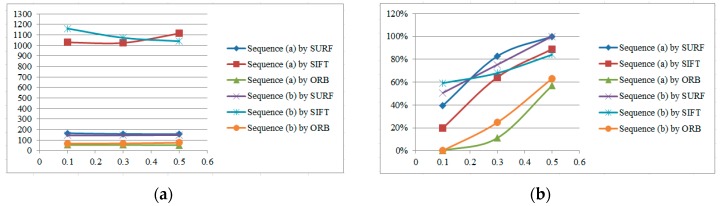
Sequence testing results for (**a**) speed and (**b**) accuracy.

We test the algorithms on a series of different images with a resolution of 640 × 480. For purposes of accuracy evaluation, 81 extra feature points are placed on the original image in a regular two-dimensional grid. Pixel intensity is interpolated at sub-pixel accuracies of 0.5, 0.3 and 0.1 pixels. Three sample images with different features are illustrated in Figure 5, where image (a) has a dark scene, image (b) has a bright object in the scene, and image (c) has several cars against a clear green outdoor scene. Results of speed and accuracy tests are listed in Figure 6. The accuracy for feature point detection is defined as the ratio of the number of detected points to 81; a point is recognized as being detected when its Euclidean distance, as calculated from the initial position, is smaller than the sub-pixel accuracy. The speed of point detection is derived from the calculation time for one image, measured in microseconds (ms). For comparison, we give an example of the outdoor scene results at 0.1 pixels (Figure 7); the other tests have similar results. At the same time, the algorithms are tested on sample images in the video sequences. Figure 8 shows video frames from the sample sequences in MATLAB’s image processing toolbox, (a) and (b) are the indoor and outdoor scenes respectively. Average values of the results of speed and accuracy tests are listed in Figure 9, for ten frames starting with the presented one in the sequences. The tests also have similar results. The results show that the SURF algorithm accurately detects the feature points in the sub-pixel space; an improvement in speed is expected from mapping onto FPGA substrates in future work.

### 2.3. Quality Assessment by Using PSNR and Trajectory Tracking

Peak signal to noise ratio (PSNR) and trajectory tracking are applied when assessing the quality of image stabilization. The PSNR is defined in Equation (4):

(4) $$PSNR\left(I_{m},I_{n}\right)=10\log\frac{255^{2}}{MSE\left(I_{m},I_{n}\right)}$$

where $I_{m}$ and $I_{n}$ refer to two frames, and $MSE\left(I_{m},I_{n}\right)=\frac{1}{MN}\sum_{i=1}^{N}\sum_{j=1}^{M}{\left(I_{m}\left(i,j\right)-I_{n}\left(i,j\right)\right)}^{2}$ refers to the mean square error of the two frames, with the values calculated by scanning through one image with *N* rows and *M* columns.

**Figure 10:**
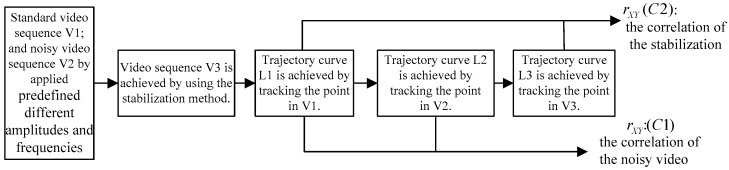
Trajectory curves and their correlation coefficients in the image sequences.

Trajectory curves are described by tracking a point in the image sequence as shown in Figure 10. The effect of the stabilization is then determined by comparing the values of the correlation coefficients $r_{XY}(C1)$ and $r_{XY}(C2)$. The correlation coefficient $r_{XY}$ of two trajectory curves is calculated by Equation (5), where (X,Y) is the coordinate data of a point on a curve, N is the total number of points, $\bar{X}$ and $\bar{Y}$ are the mean values of X and Y, respectively:

(5) $$r_{XY}=\frac{\sum_{i=1}^{N}\left(X_{i}-\bar{X}\right)\left(Y_{i}-\bar{Y}\right)}{\sqrt{\sum_{i=1}^{N}{\left(X_{i}-\bar{X}\right)}^{2}}\sqrt{\sum_{i=1}^{N}{\left(Y_{i}-\bar{Y}\right)}^{2}}}$$

The curves’ similarity is therefore greater as the coefficient $\left|r_{XY}\right|$ approaches 1, and its values are used in the following section for discussing experimental results quantitatively with a series of vibration videos of differing predefined amplitudes. Thus, the values of the correlation coefficients are calculated to adjust the reference frame in dynamic scenes. In our experiments, the threshold values are set at 0.9 for robust vibration suppression.

## 3. Experimental Results and Discussion

**Figure 11:**
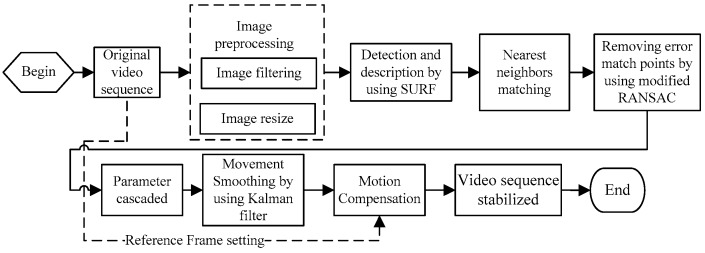
Application of the stabilization method.

This section describes the experimental results. All experiments were performed on a PC with a 3.3 GHz CPU and 4.0 GB of memory and the software was written in C++. The size of the experimental picture was 640 × 480 pixels and the size of the experimental video was 320 × 240 pixels. The process of the algorithm was applied as shown in Figure 11. Module performance testing made use of video clips of scenes of prairie and sky and sample videos captured by the in-vehicle camera. Different kinds of vibration videos captured using the mobile in-vehicle camera are discussed in the accuracy evaluations and performance assessments. Vibration video sequences of 30 fps are investigated, with increasing vehicle speeds of 20 km/h, 40 km/h and 60 km/h on stable concrete road, bumpy sand aggregate road, and soft mud road. The experimental results are provided here for mobile in-vehicle videos, in which the values of PSNR are used to estimate the quality of the image stabilization.

### 3.1. Module Performance Testing

**Figure 12:**
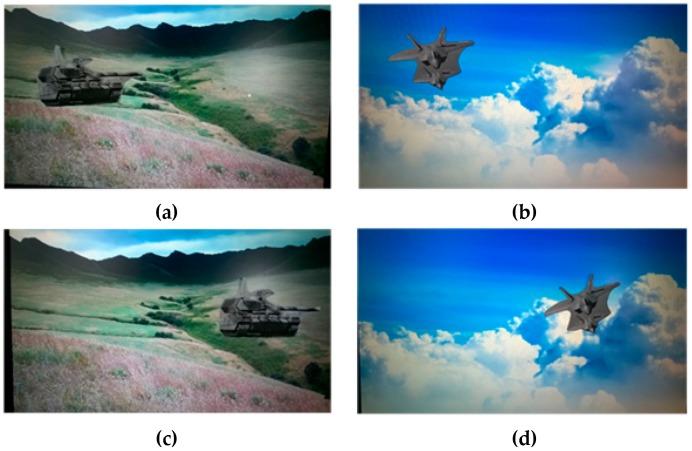
Frames in the video clips showing prairie ((**a**) and (**c**)) and sky ((**b**) and (**d**)).

**Figure 13:**
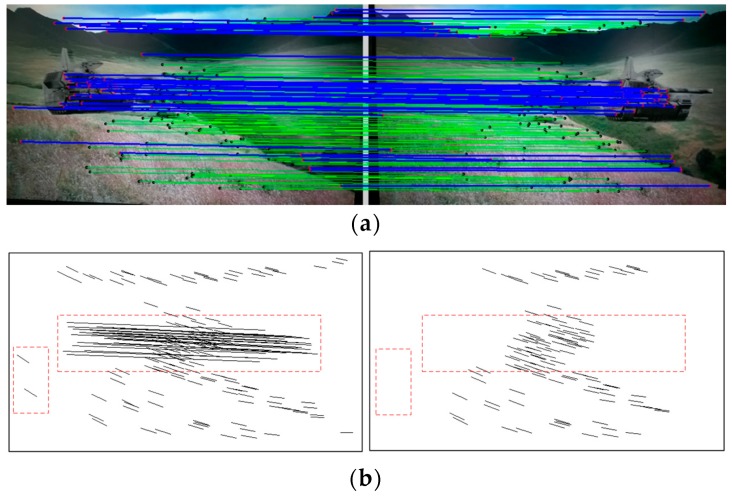
Matching pairs (**a**) and local motion vectors (**b**) for the tank target.

**Figure 14:**
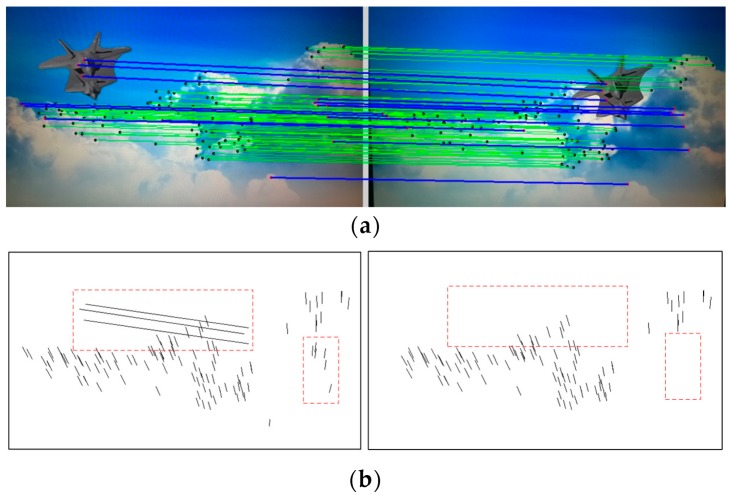
Matching pairs (**a**) and local motion vectors (**b**) for the flight vehicle target.

The module performance of the stabilization method are tested here. First, the mismatched point removal module was verified by using different kinds of video clips. Two consecutive frames in the sample video clips of the scenes of prairie and sky are shown in Figure 12, where the positions of the tank and the flight vehicle are changed, respectively. In Figure 13a and Figure 14a, green matching pairs refer to true matching, and blue pairs on the target refer to false matching on the foreground. As illustrated in Figure 13b and Figure 14b, local motion vectors between two frames were used to indicate the matching pairs, as the motion vector for false matching went in a different direction. To quantify the repeatability of the module, the affine matrices are calculated 10 times, and the results show that stable feature point matching is achieved. For the prairie video, the value of PSNR increased from 26.76 dB to 29.61 dB. For the sky video, the value of PSNR increased from 29.20 dB to 32.25 dB. The corresponding relative increases in the values of PSNR were both 10%.

**Figure 15:**
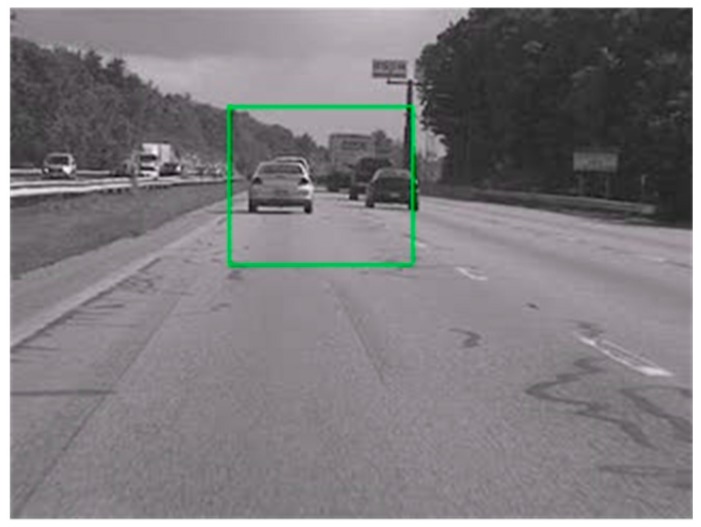
Region of interest indicated within the green box for an image sequence.

**Figure 16:**
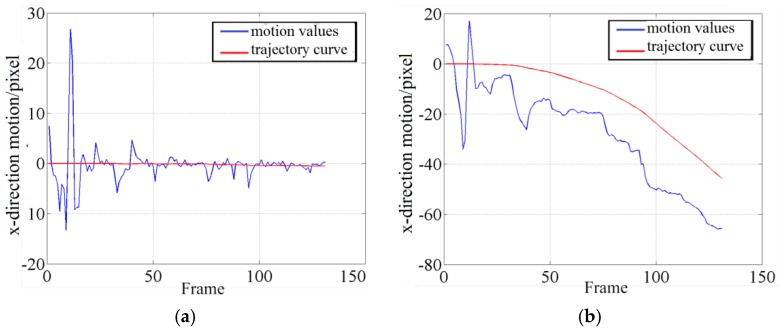
X-direction motion values between two frames in the same image sequence when (**a**) the adjacent frame and (**b**) the selected frame were used as the reference frame.

The Kalman filter is applied to the videos in connection with the modified cascading parameters by using the selected reference frame proposed in Section 2.1. One sample frame from the original video captured using an in-vehicle camera in a moving car is shown in Figure 15. The results of two different algorithm models are compared, depending on whether the modified cascading parameters were used. Figure 16 shows the x-direction motion values between two frames in the same image sequences. The models indicate that the trajectory and values of the curves in (a) and (b) varied as the reference frame changed; the blue line indicates the motion values of the video sequence and the red line is that of the trajectory curve. The values of PSNR for the region of interest in the image, as indicated by the green box, were evaluated.

**Figure 17:**
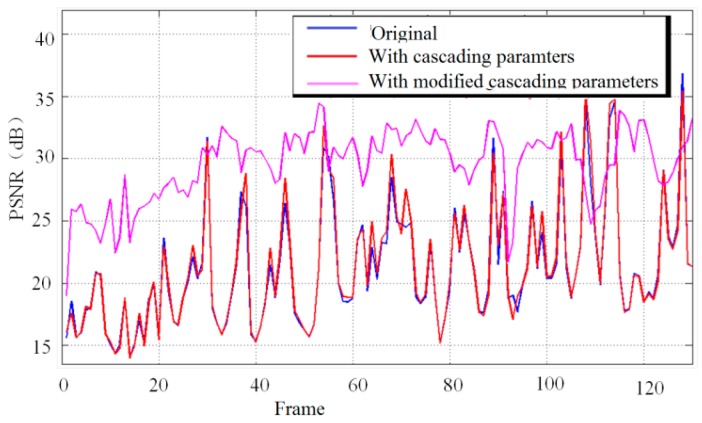
Comparison of PSNR values for the source video, the filtered video, and the filtered video with modified cascading.

As shown in Figure 17, modified cascading parameters were a precondition for achieving better image quality with a larger PSNR, by up to 8.2 dB.

### 3.2. Accuracy Evaluation with Vibration Videos of Predefined Amplitudes

**Figure 18:**
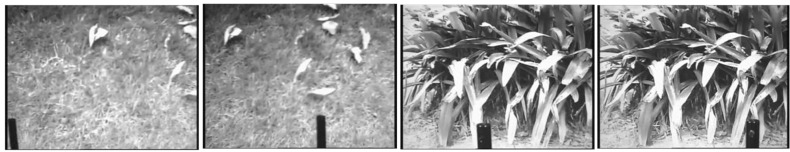
Four sample frames in the segments of mobile in-vehicle videos.

The accuracy of the image stabilization method is evaluated for the consideration of suppression variabilities. Four sample frames in vibration videos are shown in Figure 18, which are captured from a mobile in-vehicle camera. Accuracy is quantitatively calculated with the correlation coefficients, the image stabilization improving as the coefficient value approaches one. First, the background video with no vibration is captured. Second, five segments of vibration videos are captured at an amplitude of 10 Hz, with each gradually increasing as indicated by the parameter **P**, which is calculated from the ratio of the maximum vibration amplitude to the diagonal size of the image. Third, the videos are stabilized with the comprehensive algorithm. Finally, the correlation coefficients $r_{XY}(C1)$ and $r_{XY}(C2)$ are calculated, as listed in Table 1, which shows that the parameter $r_{XY}(C2)$ has the stable value of 0.9964 for the stabilized video even when the values of **P** increase to 2.09%. The results also show that the PSNR values become larger for the stabilized video in comparison to the source videos.

### 3.3. Performance Assessment Using a Vibration Video Sequence

**Figure 19:**
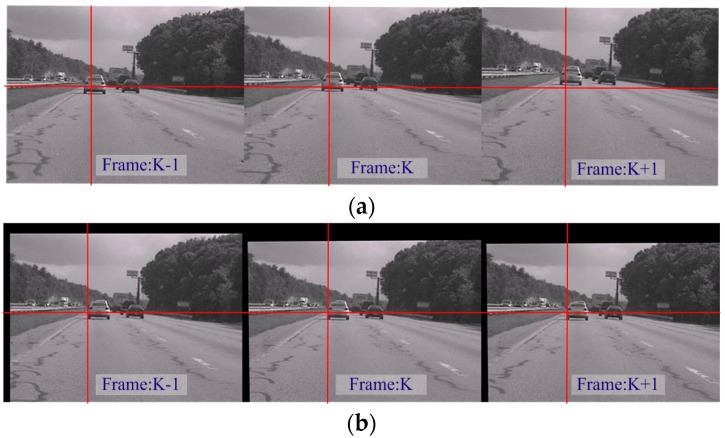
Frames in the original sequences (**a**) and stabilized sequences (**b**).

**Figure 20:**
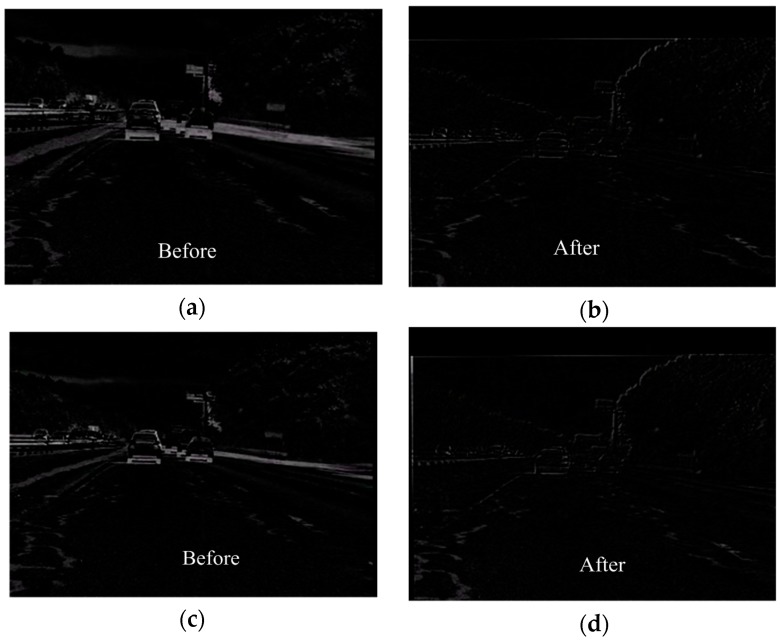
Images (**a**) and (**b**) refer to the IDI between the (*k* − 1)th and the *k*th frames in the original and stabilized sequences, respectively; images (**c**) and (**d**) refer to the IDI between the *k*th and the (*k* + 1)th frames in the original and stabilized sequences.

**Figure 21:**
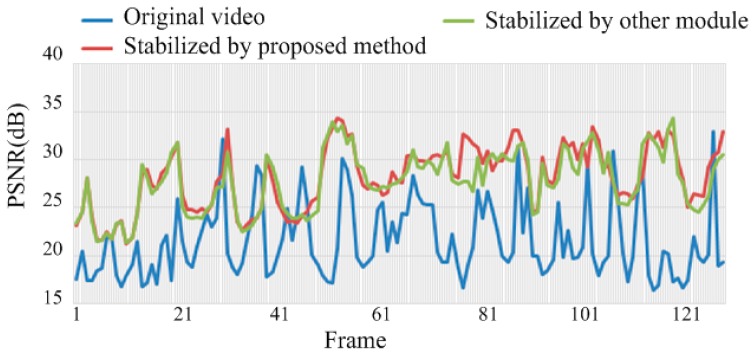
PSNR values illustrated for the original video (in **Blue**) and the stabilized video (in **Red**).

The comprehensive module of the algorithm is applied in this section, in which rotation and translation motions were included in the video. The quality of image stabilization is assessed using the values of PSNR. Consecutive frames were extracted from the original and the resulting stabilized sequences are shown in Figure 19. As indicated by the red crossed lines, the images in (a) vibrate violently in the original sequence, whereas the target images in (b) are stabilized in the new sequence. In Figure 20, the inter-frame difference images (IDIs) between frames are extracted, which shows that the profile in the stabilized sequences (b) and (d) is clearer than in the original images (a) and (c). The IDIs also help calculate the vibration amplitude, and the parameter **P** is 2.04%. Figure 21 shows the quality of the experimental video as the average values of PSNR become increasingly large. The values of PSNR for a reference method are also calculated where the Orb module is applied. The average values of PSNR for the proposed and the reference methods are 28.02 dB and 27.57 dB respectively. The average processing time for one image in the video clips is about 210 ms with the current experimental platform, with the SURF algorithm occupying about 88% of the computation time.

### 3.4. Performance Assessment Using the Video Sequences with Increasing Vehicle Speed

**Figure 22:**
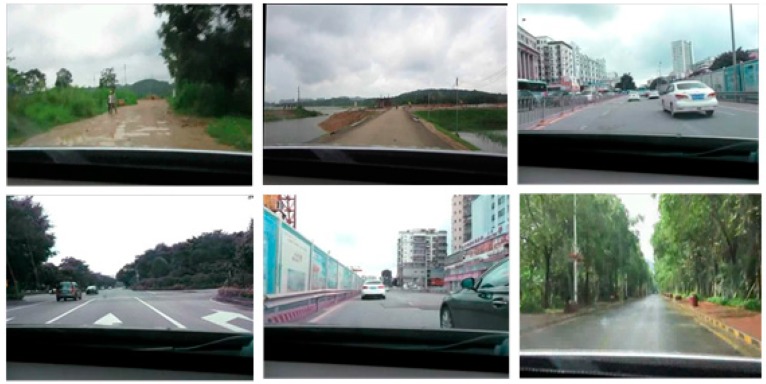
Six sample frames in the videos captured at the speed of 20 km/h, and 40 km/h.

In this section, the algorithm performance is assessed by using the vibration video sequences at 30 fps, with increasing vehicle speeds of 20 km/h, 40 km/h and 60 km/h. Six sample frames in the video sequences are shown in Figure 22. The videos were captured from a mobile in-vehicle camera when the vehicle was on stable concrete road, bumpy sand aggregate road, and soft mud road. The average processing time for one image in the video clips is about 230 ms in the current experimental platform. The experimental results proved that the target images were stabilized and the values of PSNR increased as the vehicle speed increased, as shown in Table 2.

However, the feature points in a single frame could not be distinguished when captured on the bumpy sand aggregate road and the soft mud road at the speed of 60 km/h. It is expected in future work that videos captured using a high-speed camera will have better results at 60 km/h or higher.

## 4. Conclusions and Future Work

This paper has proposed a comprehensive motion estimation technique for an improved EIS method that can be applied to a mobile in-vehicle camera. In the image sequences, correct points were extracted based on SURF and used to solve for the affine parameters. Modified RANSAC was used to purify the matching points. The Kalman filtering processes were applied to correctly compensate for motion by using modified cascade parameters. High-frequency vibration in the video sequences was effectively removed as translation, rotation, and scene scaling were taken into account. The experimental results show that the target images were stabilized using the proposed image stabilization algorithm, and the average PSNR values became increasingly large. The algorithm performance was assessed by using video sequences from the mobile in-vehicle camera, which showed the target images stabilized as the vehicle speed increased. It is expected that a high-speed camera would help achieve better results in future work. As the algorithm possesses the inherent characteristic of structural pipeline models, it can be integrated into FPGA substrates. The high-speed and super-zoom requirements of the vehicle platform will also be analyzed and integrated in future work.

## Figures and Tables

**Table 1:** PSNR values and the correlation coefficients for five segments of video.

Segment No.	P	PSNR of Source Video/dB	PSNR of Stabilized Video/dB	$r_{XY}(C1)$	$r_{XY}(C2)$
1	0.52%	22.95	24.38	0.9962	0.9932
2	0.61%	22.08	24.45	0.9900	0.9928
3	1.26%	20.60	24.40	0.9734	0.9911
4	1.40%	20.46	24.45	0.9615	0.9907
5	2.09%	20.40	24.46	0.9626	0.9964

**Table 2:** PSNR values for three road conditions at the speed of 20 km/h, 40 km/h, and 60 km/h.

NO.	Vehicle Speed/km/h	Road Condition	PSNR of Source Video/dB	PSNR of Stabilized Video/dB
1	20	Stable concrete	23.87	30.87
		Bumpy sand	23.51	25.83
		Soft mud	24.52	26.85
2	40	Stable concrete	23.46	27.28
		Bumpy sand	22.45	24.77
		Soft mud	24.34	27.32
3	60	Stable concrete	22.10	25.64
